# Analysis of predictors of rabies-positive biting animals in Cambodia using spatio-temporal Bayesian regression modelling

**DOI:** 10.1371/journal.pntd.0013478

**Published:** 2025-09-05

**Authors:** Jerome N. Baron, Yik Sik Peng, Beatriz Martínez-López, Sowath Ly, Philippe Dussart, Véronique Chevalier

**Affiliations:** 1 Center for Animal Disease Modeling and Surveillance (CADMS), Department of Medicine & Epidemiology, School of Veterinary Medicine, University of California, Davis, California, United States of America; 2 Rabies Prevention Center, Institut Pasteur du Cambodge, Phnom Penh, Cambodia; 3 Epidemiology and Public Health Unit, Institut Pasteur du Cambodge, Phnom Penh, Cambodia; 4 Virology Unit, Institut Pasteur du Cambodge, Phnom Penh, Cambodia; 5 CIRAD, UMR ASTRE, Phnom Penh, Cambodia; 6 ASTRE, Univ Montpellier, CIRAD, INRA, Montpellier, France; Tufts Medical Center, UNITED STATES OF AMERICA

## Abstract

Cambodia is endemic for rabies, a fatal zoonotic viral disease transmitted through dog bites. The Institut Pasteur du Cambodge through its Rabies Prevention Center is the main institution in charge of rabies prevention and surveillance in the country. Its main tool for prevention is post-exposure prophylaxis (PEP) for bite victims. Allocation of specific PEP regimen is done based on the assessment of the severity of the wound and the information collected by IPC doctors from patients regarding the attack’s characteristics and the attacking animal’s health status. Furthermore, a small proportion of patients bring animals for testing, 60% of which were tested positive for rabies. Using the data collected from patient interviews from 2000 to 2016, we used a Bayesian spatio-temporal regression model to identify predictors for two outcomes: i) a patient bringing an animal for testing and ii) an animal testing positive for rabies. The ultimate aim of the analysis was to provide information that could help with allocation of PEP resources. Notably non-owned animals, a large number of bite victims, and unprovoked attacks were all predictive of a positive test. A suspected rabies status assigned by doctor based on animal symptom description was also highly predictive of a rabies test, with 94.6% of tested animals that were assigned as sick being positive for rabies. Furthermore, we identified three Provinces of Cambodia with higher odds of positive tests: Kampong Cham, Kandal and Kampong Thom. This information could help allocate limited PEP resources, though this study showed IPC already a strong protocol to identify patients exposed to a rabies suspect dog.

## Author summary

Rabies is a fatal viral disease that affects the nervous system. It causes thousands of deaths in humans yearly, mostly in low-income countries in Asia and Africa where it is mainly circulated and transmitted by free-roaming dogs. The tools to prevent rabies (pre and post-exposure prophylaxis - PrEP and PEP respectively) are very effective in both humans and dogs, however lack of resources means these are often insufficiently used in endemic countries or inaccessible to large portions of the population. Cambodia does not have a dog vaccination program, but has a program to offer PEP to dog bite victims managed by the Institut Pasteur du Cambodge (IPC) through three rabies prevention centers. In this program, biting animals are also tested for rabies by IPC when available. Using IPC patient and animal testing data, and statistical modelling we aim to identify features that predict a rabies positive animal to guide PEP resource allocation, identify provinces with higher risk of rabid animal to guide future openings of PEP centers, and identify the selection bias for the animals tested.

## 1. Introduction

Rabies is a fatal zoonotic viral disease which can infect most mammalian species and is responsible for the deaths of nearly 60,000 people per year globally and billions of dollars in costs due to health expenses and loss of livestock [1–3]. The vast majority of human deaths result from exposure through dog bites in developing countries in Africa and Asia where dogs are typically free-roaming [1]. Once symptoms appear, rabies is nearly always fatal [4,5]. Cambodia, like most countries in Southeast Asia, is endemic for canine mediated rabies. It is estimated that, in Cambodia, around 800 people die of rabies each year, giving it one of the highest rates in the world at 6 per 100,000 [6]. Cambodia has a relatively large owned but mostly free-roaming dog population with dog-to-human ratios ranging from 1 dog for 2.8 people to 1 dog for 4.8 people [6–9]. Survey studies have reported bite rates of 1.1 to 4.8 bites per 100 person-years depending on the province, which was extrapolated as 375,000–600,000 bite injuries nationally per year [7,9,10].

Despite the prospective negative outcome following a bite, post-exposure prophylaxis (PEP) has proven a very effective tool in preventing disease, before symptoms start [11]. The PEP regimen used at IPC consists of the immediate administration of rabies immunoglobulins (RIG) followed by a condensed 1-week 2-sites intradermal vaccination regimen over 3 visits, as recommended by WHO [11–14]. RIG is not always allocated and is dependent on the severity of the wound or the risk profile of the attack. In Cambodia, until 2018, three non-private entities offered PEP: the Pasteur Institute of Cambodia (IPC) and the National Institute of Public Health clinic in Phnom Penh, and the Angkor Hospital for Children in Siem Reap [15,16]. Together these centers administered some 25,000 PEP regimens per year, 85% of which were at IPC [16,17]. Some private clinics also offer PEP across Cambodia, but the number of doses and the reliability of both treatment protocols and injected substances are not confirmed. IPC started distributing PEP in 1995, initially for free and with a fee since 2010 amounting to US$ 12 for a full vaccine course and US$ 37 for RIG [15,16]. In order to expand PEP capacities, IPC opened two new centers, a first in Battambang Province in July 2018 and a second in Kampong Cham Province in March 2019 [18,19]. However, concurrent to these openings, in February 2019, a video of a young Cambodian girl dying of rabies was widely distributed on social media leading to a surge in demand for PEP [20,21]. In 2019, IPC recorded nearly 78,700 PEP patients, stretching limited PEP resources beyond normal capacity and causing long waiting times for patients [22]. This exemplifies how limited PEP resources can quickly be overwhelmed. Despite this, the number of patients coming to IPC remains much lower than the estimated number of dog-bites in Cambodia even when considering treatments provided outside, and thus unrecorded. This is further exacerbated by the unbalanced geographical coverage linked to accessibility, given the limited number of locations offering reliable PEP [23]. This demonstrates the need for increased communication and access to PEP, and the ability to identify bite victims most at risk of rabies to better allocate limited supplies of RIG and vaccine.

When bite patients present to IPC, an initial assessment of rabies risk is done on arrival through a questionnaire focusing on the characteristics of the injury, the attack, and the animal perpetrating it. Patients exposed to animal saliva will all receive the vaccine; however, RIG is only allocated based on wound severity and when the animal is suspected of being rabid. When available, the animal head is tested for rabies using direct fluorescent antibody test (FAT) to confirm suspicion. The geographical location of the attack is collected. Human rabies cases are however rarely recorded. With just 1.5% of PEP patients bringing an animal for testing, only a few hundred heads are tested every year with around 60% of them being positive [6]. Through statistical modelling, we can identify patient, animal, and attack characteristics that are predictive of a patient bringing an animal for testing and of an animal testing positive for rabies with the aim of 1) raising awareness in the population on the risk of getting rabies after an animal bite, and 2) guiding PEP resource allocation to patients that are at greater risk. However, estimating the true burden of canine rabies would require population level field investigations such as serological surveys or active contact tracing, beyond the testing provided by the passive surveillance system in place in Cambodia and the analysis performed in this study [24,25]. Nevertheless, we can investigate the geographical risk of exposure in patients and identify higher-risk areas by taking into account spatio-temporal distribution and auto-correlation of tested animals in the model structure. Studies investigating risk factors of animals testing positive for rabies or on demographic characteristics of PEP patients of have been conducted in Thailand and Vietnam [26–28], but using a spatio-temporal Bayesian framework with a spatial-autocorrelation matrix we can estimate a more complete geographical distribution of exposure risk [29]. Furthermore, none of these studies investigated in conjunction how the same factors might be associated with both the selection of tested animals and the test result, leading to a potentially unaccounted bias in the sample of tested animals.

The goals of this study are threefold. First, we aim to quantify bias in the selection of tested animals by identifying predictors of a patient bringing an animal for testing. This can also serve as a proxy for rabies awareness, knowledge, and perceptions in bite wound patients. Second, we aim to identify predictors of a biting animal testing positive for rabies in order to inform on the individual exposure risk of patients presenting for PEP. Third, in addition to a study seeking to predict PEP needs geographically [23], we aim to identify high-risk areas in Cambodia where future PEP centers would be most beneficial and where canine vaccination campaign efforts should be focused.

## 2. Methods

### Ethical statement

All personal and identifiable information was removed prior to analysis so as to maintain patient confidentiality. Ethical approval was not required as data were de-identified and aggregated at the provincial level.

### 2.1. Data collection and laboratory diagnosis

Data for the years 2000–2016 were provided by IPC. When patients present at IPC for PEP, a document is completed in four main sections. Three of these concern (i) patient characteristics (2 variables modelled), (ii) characteristics of the exposure and wound (12 variables), and (iii) animal characteristics (4 variables) (S1 File, S1 and S2 Tables). For these three sections, all questions in the survey were considered for analysis as this was an exploratory analysis, however a number of variables were rejected for the following reasons: variables contained identifiable information (address and coordinates), variables had a majority of missing answers (pregnancy status, nationality, presence of bleeding in the wound, presence of sutures in the wound), variables were co-linear to the outcome (status of the animal after the attack). This last variable contained four categories (killed, dead, disappeared, healthy) was not included as tested animals were exclusively killed or found dead, whereas healthy (which means alive) and disappeared animals were exclusively not tested. The fourth section includes information on prior vaccination history and the progress of the current PEP regimen, which is completed during follow-up visits. This section was not considered for analysis as it relates to the care of the patient after the exposure and is posterior in the causal pathway. In the animal characteristics section, a question on the animal’s health appearance is completed by the doctor based on a set of secondary questions focusing on specific symptoms, the status of the animal post-attack, the spontaneity of the aggression, and the number of victims. This aggregate question is used to determine if a dog is suspected rabid and guides the allocation of RIG at the start of the PEP regimen (S2 File). Between 2000 and 2016, 9841 patients out of the total number of patients seeking PEP (3.3%) received RIG, ranging from 0.8% in 2005 to 7.5% in 2014.

When patients bring the head of the attacking animal, this head is systematically tested at IPC using FAT [6,30]. This method uses polyclonal or monoclonal FITC-conjugated antibodies to detect the presence of viral antigens in brain tissue with fluorescence microscopy. It can only be performed post-mortem. It is the WHO and OIE recommended gold standard test with sensitivity and specificity nearing 99% [31,32].

### 2.2 Data management

Inconclusive tests were excluded from analysis of test results. Predictor variables followed five broad groups, and the same predictors were used in both analyses. The first group of variables involved the characteristics of the victim, i.e., sex and age groups. The third group described the characteristics of the exposure: including the province in which the attack took place, then year, month and number of days from accident to consultation. Cambodia’s administrative divisions have changed significantly during our study period, including the division of Kampong Cham province in 2013, creating the new 25^th^ province of Tboung Khmum. For this study, we applied the provincial boundaries prior to 2013 for the whole time period, keeping the number of provinces at 24, and adding Tboung Khmum data to Kampong Cham. This group also included variables on the exposure type (binary: bite or other such as scratch and licking contact) and number of known victims. The fourth group was the characteristics of the wound: health status of the surface exposed, severity of the wound, location of wound, and number of wounds. Severity of the wound was defined as a bite that would fall within the WHO rabies exposure of category III: single or multiple transdermal bites or scratches [33]. The last group included characteristics of the animal: species (dog, cat, other domestic, and wildlife), spontaneity of aggression (provoked or not), health appearance (healthy or sick) and ownership status (with or without owner). Species was defined, from 2000 to 2012 in the four categories above, with no more details being recorded. From 2013 to 2016, other species were more detailed, and the “other domestic” was combined with pigs, cattle, buffalos, horses, and rabbits. Whereas “wildlife” was combined with primates, bats, ferrets, civets, reptiles, non-poultry birds, bears, and large felines. As a note, domestic and wild is defined here on a species basis, and a number of animals categorized as wildlife in the data were marked as owned animal (captive or domesticated wildlife), whereas a number of animals from domestic species were not owned (feral animals).

A number of variables were categorized as these were strongly skewed, with a few outliers. Age was categorized into four groups: small children (0–9 years), children and adolescents (10–19 years), adults (20–59 years), and older adults (60 and above). The highest recorded age was 88. The number of lesions ranged from 0 to 23 with a median of two, and it was decided to categorize it into five groups (1, 2, 3, 4, 5 or more lesions). Similarly, the number of victims ranged from 1 to 15 with a median of one, and it was decided to categorize it into four groups (1, 2 or 3, 4 or 5, 6 or more victims). Finally, time from accident to consultation ranged from 0 to 59 days, with a median of one and was categorized into 1 day, 2 or 3 days, 4 or 5 days and 6 or more days.

For wound location, the data initially recorded nine different locations. However, numerous patients had multiple wounds in different locations, leading to a large number of different mixed location categories. To account for this, wound location was divided into five dummy binary variables: head & neck, trunk & genitals, arm, hand & fingers, leg, and foot.

A number of patient records were incomplete for the variables included in modelling. These are included in the general data descriptions, but are excluded from the statistical analysis and no imputation was used.

### 2.3. Statistical analysis

Two binary outcomes were used: a patient bringing an animal for testing (outcome 1), and a tested animal being positive for rabies (outcome 2). Exploratory univariate analysis was done using generalized linear mixed model (GLMM) with the R package “lme4” [34]. These univariate analyses were used to explore the relationships between each potential predictor and the two outcomes, and guide categorization of continuous variables. Province was included as a random intercept. The Akaike Information Criterion (AIC) was used to determine if continuous or categorized variables were more appropriate for the following multivariate model selection.

To be able to incorporate conditional auto-regressive structures for the spatial random effect as well as time, a Bayesian framework using Integrated Nested Laplace Approximations (INLA) was used for our multivariate model building. This method has the benefit of generating a random effect estimate and posterior probability for provinces without any data-points, thanks to the correlation with neighboring provinces though with high uncertainty. Month was used as a cyclic auto-correlated random effect to capture the potential impact of seasonality. Equal weight was assigned to all contacts between provinces. Default non-informative priors were used in this model. Default priors for the hyperparameter Ƭ of the temporal random effect follows a Log-Gamma distribution with parameters of 1 and 0.00005. For the hyperparameters of the spatial random effect, default priors are a Penalized Complexity (PC) prior with parameters 1 and 0.01 for Ƭ1 and a PC prior for precision with parameters 0.5 and 0.5 for Ƭ2. Year was also tested as a non-cyclic auto-correlated random effect but was eventually included in the model as a simple non-correlated random effect as there was no evidence of yearly temporal trends in the proportion of positive tests. Model selection was performed with a two-way stepwise selection process from an empty model using the Deviance Information Criterion (DIC) and the Watanabe-Akaike Information Criterion (WAIC). Data analyses were done using the package R-INLA [35–38].

### 2.4. Fit and validation

For all multivariate Models, we obtained fitted probabilities for an animal being tested (Model 1) or testing positive for rabies (Models 2). The R package “pROC” was used to generate Receiver Operating Characteristic curves (ROC curves) to evaluate the models’ predictive performance and select an optimal prediction probability threshold [39]. The area under the curve (AUC) was generated to assess how predictive each model was as a whole. Then, the optimal prediction threshold was selected by maximizing the sum of sensitivity and specificity using Youden’s J statistic [40]. The prediction threshold is the value above which a fitted probability is considered a positive result and below which it is considered a negative result. For the selected threshold, we produced model performance statistics, including Sensitivity, Specificity, Positive Predictive Values (PPV) and Negative Predictive Values (NPV). PPV and NPV represents the probabilities of a positive and negative prediction from the model being truly positive and negative in the test result respectively.

As the dog “health appearance” variable used in model 2 had a very high odds ratio, it was decided to calculate predictive values for it as if it were a test result, with the actual test result being the gold standard. In this case, the category “healthy” was considered negative result, and the “sick” a positive result. This was done to compare the predictive performance of that variable alone against that of model 2.

The fitted values from Model 1 were also used in a ROC curve analysis on the outcome from Models 2. This was done to investigate how predictive Model 1 was, not only the probability of an animal being tested, but of that test returning positive as well. The aim here was to provide evidence of potential bias in the sampling.

All analysis and data management was done in R version 4.0.3 [41].

## 3. Results

### 3.1. Descriptive

**Fig 1:**
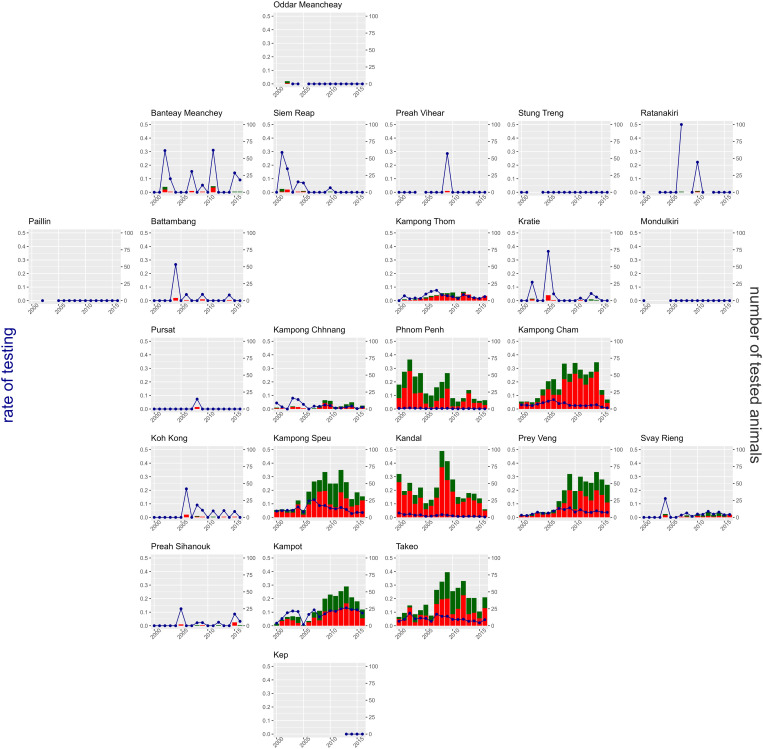
Number and rate per patient of tested animals by province and year in Cambodia from 2000 to 2016. Years in which there are no testing rate points are years in which no patient from that province presented at IPC for PEP. Provinces are presented in a geographical approximation, to highlight the similarities between neighboring provinces.

In total 294,040 patients came to IPC Phnom Penh for PEP from 2000 to 2016. Of these, 415 incomplete patient records for the variables modelled were excluded from analyses, leaving 293,625 observations for model 1 selection. Details for missing answers of specific variables are given in S1 and S2 Tables. Of all the patients, 4,515 (1.5%) brought an animal for testing. Six test results were inconclusive and discarded from the analysis for the second outcome and seven patients had incomplete records, leaving 4,502 test results to be analyzed for models 2A and 2B, 60.5% (2,724) of which were positive. Of the tested animals, 98.1% (4,428) were dogs, 60.7% (2,686) of which were positive. Seven of Cambodia’s 24 provinces accounted for 93.0% (4,198) of tested animals (Fig 1). These were concentrated in close proximity to the capital Phnom Penh, where IPC is located with numbers ranging from 443 to 728 tested animals. These seven provinces contained 92.5% (2,521) of positive animals and 95.7% (281,490) of PEP patients. However, the rate of testing varied greatly between them, with 9.9% of patients coming from Kampot Province bringing an animal for testing compared to 0.3% in Phnom Penh. The percentage of positive animals varied from 52% to 56% in five of these seven provinces, whilst the provinces of Kandal and Kampong Cham had noticeably higher values at 72% (526/728) and 73% (474/653) respectively. In 13 provinces, the number of tested animals varied from two to 106 with wider ranges of positive rates from 25% to 100% (S2 Table). Finally, four provinces, totaling 132 patients, had no animals tested for the period 2000–2016.

**Fig 2:**
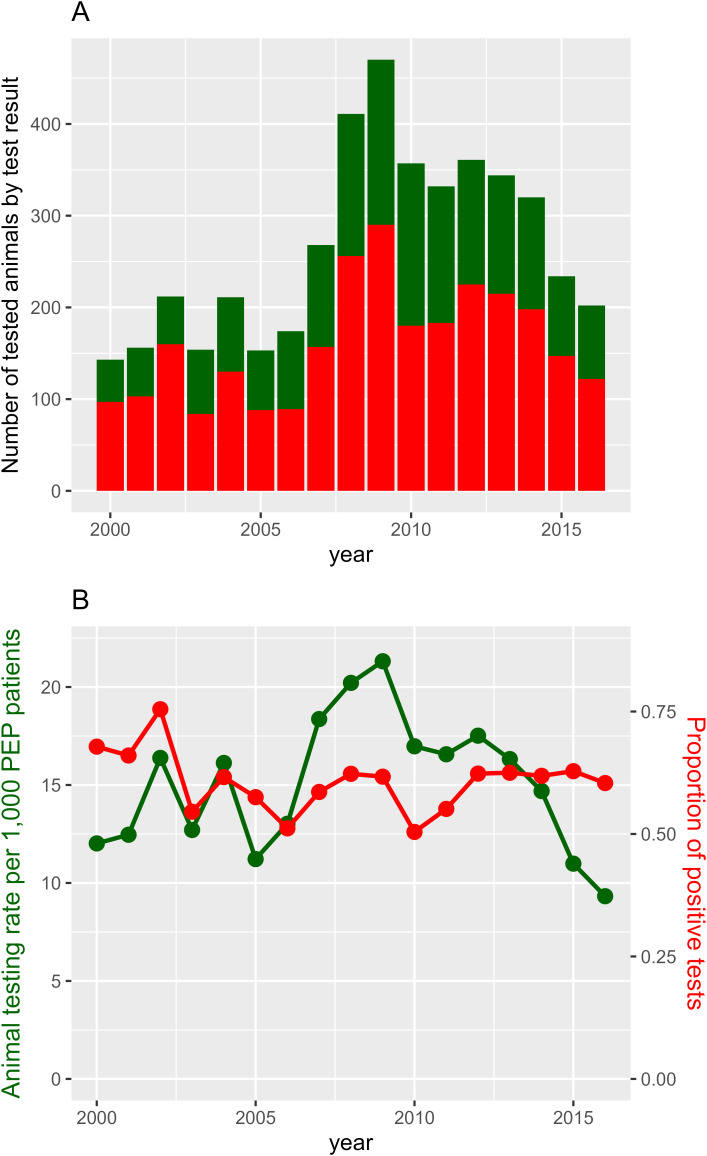
Counts (2A) and rates (2B) of animals tested and positive for rabies in Cambodia from 2000 to 2016. Red lines represent the rate of testing amongst patients and green lines the proportion of positive tests.

The number of tested animals was relatively stable from 2000 to 2006, ranging between 143 and 212 yearly. It then increased quickly from 2007 to a peak of 471 in 2009 before gradually dropping back to 202 by 2016. This peak is particularly noticeable in Kandal Province as can be seen in Fig 1. The proportion of positive dogs remained stable over time, however, with 11 of 17 years having values between 58% and 63%, and the rest ranging from 50% to 75% (Fig 2).

### 3.2. Model 1 results

Numerous variables proved to have a significant association with both outcomes in univariate models. As most of these are described later in the multivariate results and had similar results, only major differences with multivariable results will be described.

In Model 1, we selected three animal related variables (health appearance, spontaneity of aggression, and species), seven attack and wound related variables (type of exposure, number of victims, number of lesions, severity of wound, and three locations: head & neck, hand & finger, and leg), one demographic variable (age category).

Detailed results for Model 1 are presented in Table 1. Animals noted as appearing sick had much higher odds of being tested compared to animals noted as healthy (OR=77.73, CI = 71.66 to 84.32). Provoked aggressions led to lower odds of testing (OR=0.86, CI = 0.79 to 0.94), and any species other than dogs also had lower odds of being tested (ORs ranging from 0.27 to 0.59). This is different from the univariate where livestock showed higher odds of testing compared to dogs. Bites were much more likely to lead to testing compared with other types of exposures having an OR of 0.01 (CI = 0.01 to 0.02). An increasing trend in ORs was noticeable as the number of victims from an attack increased (OR from 1.64 to 2.08) and as the number of lesions increased (OR from 1.18 to 2.63). Severe wounds (OR=1.40), wounds to the head and neck (OR=1.33), and wounds to the hands and fingers (OR=1.19) all had higher odds of being tested, whereas wounds to the leg led to lower odds (OR=0.93), though the latter had a CI that includes one. Finally, we observed decreasing odds of an animal being tested as age groups increased relative to the young children of nine years or less group (ORs from 0.63 to 0.85).

**Table 1:** Results from multivariate model 1: predictors of an animal being tested after a bite incident with a patient seeking PEP at the IPC in Phnom Penh. Counts presented differ from S1 and S2 Tables as they represent the subset analyzed, excluding incomplete patient records.

Variable	Category	Number of Patients	Number of tested animals	Percentage of tested animals	Odds ratio (Model 1)	95% credibility Interval
Total	NA	293,625	4,508	1.54		
**Animal**						
Animal health appearance	Healthy	285,816	1,786	0.62	Ref	
	Sick	7,799	2,722	34.90	77.73	71.66 to 84.32
Aggression	Spontaneous	195,546	3,401	1.74	Ref	
	Provoked	98,079	1,107	1.13	0.86	0.79 to 0.94
Animal species	Dog	277,463	4,422	1.59	Ref	
	Cat	12,742	45	0.35	0.37	0.27 to 0.52
	Livestock	766	34	4.44	0.59	0.38 to 0.90
	Wild	2,648	7	0.26	0.27	0.12 to 0.58
	Other*	6	0	0.00	NA	NA
**Victim**						
Age categories	0 to 9 years	104,888	1,728	1.65	Ref	
	10 to 19 years	62,308	980	1.57	0.85	0.77 to 0.93
	20 to 59 years	112,255	1,615	1.44	0.73	0.67 to 0.80
	60 years or more	14,174	185	1.31	0.63	0.52 to 0.76
**Attack and wounds**						
Type of exposure	Bite	292,226	4,494	1.54	Ref	
	Other	1,399	14	1.00	0.01	0.01 to 0.02
Number of victims	1	231,546	2,443	1.06	Ref	
	2 or 3	48,460	1,431	2.95	1.64	1.51 to 1.79
	4 or 5	10,106	383	3.79	1.58	1.37 to 1.83
	6 or more	3,513	251	7.14	2.07	1.73 to 2.50
Number of lesions	1	48,546	539	1.11	Ref	
	2	221,533	3,260	1.47	1.18	1.04 to 1.33
	3	16,589	426	2.57	1.43	1.21 to 1.71
	4	4,790	172	3.59	2.08	1.65 to 2.63
	5 or more	2,167	111	5.12	2.63	1.97 to 3.51
Severity of wound	Not severe	246,900	3,378	1.37	Ref	
	Severe	46,725	1,130	2.42	1.40	1.26 to 1.54
Wounds on head and neck	No	275,708	4,107	1.49	Ref	
	Yes	17,917	401	2.24	1.33	1.15 to 1.53
Wound on hands or fingers	No	236,282	3,378	1.43	Ref	
	Yes	57,343	1,130	1.97	1.19	1.08 to 1.31
Wound on legs	No	211,908	3,514	1.66	Ref	
	Yes	81,717	994	1.22	0.93	0.85 to 1.02

*Other: refers to individuals seeking PEP after exposure to human patients or pre-exposure prophylaxis.

**Fig 3:**
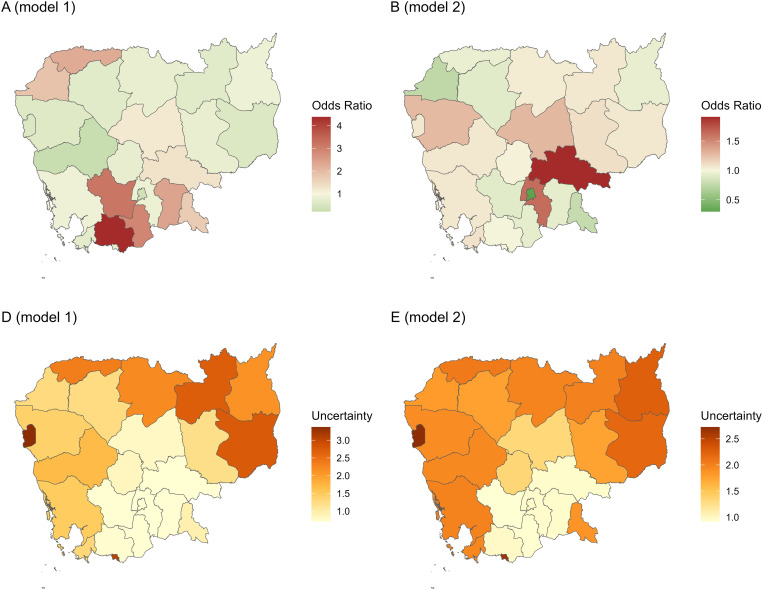
3A, 3B, 3C and 3D: Maps of provincial odds ratios and uncertainty for the Bayesian spatio-temporal logistic regression models. (A) Odds ratio for having an animal tested in model 1 (B) Odds ratios for testing positive in model 2. (C & D) Uncertainty for the odds ratios in map A & B respectively. Uncertainty is calculated as the difference between the 97.5 percentile and the 2.5 percentile of the non-exponentiated coefficients for each province. The base layer for this map was obtained from GADM version 3.6: https://gadm.org/old_versions.html. Details for the corresponding license can be found at: https://gadm.org/license.html.

When adjusted for other variables in the model, Kampot, Kampong Speu, Takeo, and Prey Veng stand out as having higher odds of testing amongst the 10 provinces with more than 1,500 PEP patients, and with the lowest uncertainty in the estimates (Fig 3A and 3D and S3 Table). The remaining provinces (Kampong Thom, Kampong Chhnang, Kampong Cham, Kandal, Phnom Penh, and Svay Rieng) all have odd ratios close to or lower than one. Despite smaller number of patients and so higher uncertainty, the provinces in the North West (Battambang, Pursat, and Siem Reap) had some of the lowest odds of having animals tested.

### 3.3. Model 2 results

Model 2 looks at predictors of rabies positive animals, including the animal health appearance variable. I We selected four biting animal related variables (health appearance, spontaneity of aggression, ownership status, species), two attack and wound related variables (number of victims, number of lesions), and finally month and year of the incident as random effects. Detailed results are presented in Table 2. Animals designated as appearing sick had significantly higher odds of being rabid compared to healthy ones (OR=460.70, 95% CI = 328.11-649.12). To a lesser degree, feral or wild animals also had significantly higher odds of testing positive compared to owned animals (OR=19.35, 95% CI = 9.94-37.68).

**Table 2:** Results from multivariate models 2: predictors of an animal brought by a PEP patient to IPC, testing positive for rabies. Counts presented differ from S1 and S2 Tables as they represent the subset analyzed, excluding incomplete patient records.

Variable	Category	Number of tested animals	Number of positive animals	Percentage Of positive animals	Odds ratio (Model 2)	95% credibility Interval
Total	NA	4,502	2,724	60.5	NA	NA
**Animal**						
Animal health appearance	Healthy	1,783	150	8.4	ref	–
	Sick	2,719	2,574	94.7	460.70	328.11 to 649.12
Aggression	Spontaneous	3,400	2,188	64.35	ref	–
	Provoked	1,102	536	48.6	0.31	0.23 to 0.43
Animal ownership	Owned	4,031	2,274	56.4	ref	–
	Feral or wild	471	450	95.5	19.35	9.94 to 37.68
Animal species	Dog	4,416	2,684	60.8	ref	–
	Cat	45	12	26.7	0.06	0.02 to 0.16
	Livestock	34	27	79.4	0.80	0.21 to 3.13
	Wild	7	1	14.3	0.11	0.00 to 20.13
**Attack and wounds**						
Number of victims	1	2,438	1,225	50.3	ref	–
	2 or 3	1,430	981	68.6	1.86	1.40 to 2.48
	4 or 5	383	304	79.4	3.75	2.26 to 6.20
	6 or more	251	214	85.2	4.26	2.17 to 8.39
Number of lesions	1	538	335	62.3	ref	–
	2	3,255	1,967	60.4	1.53	1.00 to 2.33
	3	426	265	62.2	1.22	0.68 to 2.17
	4	172	103	59.9	1.43	0.68 to 3.03
	5 or more	111	54	48.7	0.41	0.16 to 1.02

Conversely, provoked aggressions were significantly less likely to be associated with a positive rabies test compared to spontaneous ones (OR=0.31, 95% CI = 0.23-0.43). Cats had lower odds of testing positive compared to dogs (OR=0.06, 95% CI = 0.02-0.16). Interestingly, wild animal species had significantly lower odds of being positive in the univariate model (OR=0.10, 95% CI = 0.01-0.81), but this became non-significant in the multivariate.

For the attack related variables, the odds of positive test increased with the number of victims, ranging from an OR of 1.86 with two or three victims to 4.26 with six or more victims compared to the reference of one victim, with all ORs being significantly different from the reference. For the number of lesions, two, three or four lesions had similarly higher odds of positive test compared to one lesion but none of these were significant. This differed from the univariate model where having two, three, or four lesions all had non-significant odds ratios just below one. The five or more lesions category had lower odds of a positive test but this was non-significant in Model 2A despite being significant in the univariate model (OR=0.52, 95% CI = 0.34-0.80).

When looking at results for the spatial random effect, we observed that Kandal and Kampong Cham had higher odds of positive tests that were much above average, with Banteay Meanchey, Phnom Penh and Svay Rieng having the lowest values (Fig 3B and S3 Table). Unsurprisingly, the uncertainty levels are lowest in the seven provinces with most tested animals as described in section 3.1. (Fig 3E). Kampong Thom and Kampong Chhnang also had higher numbers of tested animals (106 and 71, respectively) and have relatively low uncertainty compared to the rest of the provinces where data were scarce.

Given the very high impact of the animal health appearance variable on model 2, it was decided to rerun model selection after removing this variable from the process to investigate other potential variables that might be associated with test results (S4 Table). This led to the addition of seven more variables than the ones already described above: sex and age of patients, time from accident to consultation, and four wound locations (hand and fingers, arms, legs, and trunk and genitals). Briefly, women had lower odds of positive rabies tests compared to men whereas adult age groups had higher odds compared to children under age nine. Bites at the hands and arms had higher odds of a positive tests whereas bites to the legs and trunk had lower odds. Finally, longer waiting times from accident to consultation where associated with lower odds of positive tests.

### 3.4 Model fit

**Fig 4:**
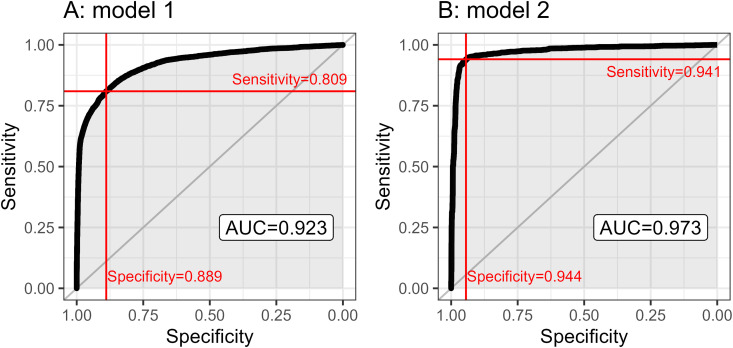
4A, 4B and 3C: ROC curves for the INLA models 1 (A), 2 (B). The black lines represent the ROC curve, and the red lines are the selected values for sensitivity and specificity at the peak Youden’s J.

For Model 1, the area under the curve (AUC) was very close to 1 (0.923) with a ROC curve close to the top left corner, suggesting strong predictive abilities of the model (Fig 4A). With the selected prediction threshold, this model’s specificity and sensitivity were high, 0.89 and 0.81 respectively. With these model performances and due to the very low rate of animal testing amongst PEP patients, the model yielded a very high negative predictive value (0.997) but a very low positive predictive value (0.10).

Model 2 appeared highly predictive, with an AUC above 0.97, which is clearly illustrated with the ROC curve being nearly at a right angle in the top left corner (Fig 4B). Accordingly, Model 2 showed strong test performances once a probability threshold was selected with specificities and sensitivities close to 0.94. Similar observations can be made with the predictive values, confirming the highly predictive nature of this model.

For comparison, the most influential variable in the model (animal’s health appearance as assessed by IPC doctors based on the victim’s description of symptoms) was used as a simple binary test and compared to the actual test results. This yielded test performances that were very close to Model 1, with a similar sensitivity (0.945), but a slightly lower specificity (0.916).

### 3.5. Comparison of models 1 and 2

Using ROC curve, we also tested how well the fitted values from Model 1 fitted the output used in Models 2. Performance statistics of Model 1 were very similar to model 2 (Fig 4), meaning that the set of variables that were predictive of a biting animal being tested was also strongly predictive of this animal testing positive. Indeed, numerous variables (animal health appearance, spontaneity of aggression, species, age, number of victims, number of lesions, and location of lesions) were in common between Models 1 and 2, and the direction and scale of effect are often similar. This is particularly noticeable with the variable having the strongest effect in each model: animal health appearance. However, aggression, animal species (for cats and wildlife), wounds located in upper body (head and neck, hands and fingers, arms), and number of victims were other variables predictive both of the animal being tested, and of the animal testing positive. Notably, though animal ownership was not selected in Model 1, it was significantly associated with testing an animal at the univariate level, with the same direction of effect observed in Models 2. Overall, this suggest that the selection of animals for testing is biased towards animals at higher risk of rabies.

However, other variables predictive of testing a biting animal were not predictive of this animal being positive. For instance, livestock, despite having higher odds of being positive, had lower odds of being tested. Similarly, when victims were from older age groups, the odds of having the animal tested were lower, but the odds of a positive test were higher. A larger number of lesions (3 or more) had higher odds of leading to a test, but was not significantly associated with the test positive result. Finally, the severe wound was associated with higher odds of animal testing but was not selected in any of the test result models, nor was it significantly associated with the test result at the univariate level.

## 4. Discussion

### 4.1. General observations

From 2000 to 2016, the number of PEP patients presenting at IPC gradually increased. This was initially accompanied by an increase in animal testing, peaking in 2007–08 when a study to improve rabies diagnostics was set up by IPC, before dropping again. However, during this time period, the proportion of animals testing positive remained relatively constant, around 60%. This is similar to what was observed in neighboring Thailand, which saw stable and high rates of rabies positive animals over long periods of time, even as the number of tested animals dropped [26,42]. This provides strong evidence that rabies remains endemic and a serious public health concern in Cambodia, and the broader region. [43]. With limited data on human rabies cases in Cambodia, animal testing, even if based on voluntary reporting, is an important indicator of disease presence and evolution. However, we should note that these data predate important developments such as the opening of new centers in Battambang (2018) and Kampong Cham (2019), which will likely improve the spatial coverage of data collection [18,19]. Further, the posting on social media of a video of a young girl dying from rabies in 2019 led to a rapid increase in patients, followed a few months later by the COVID pandemic which had the opposite effect (S5 Table) [20,21,23].

### 4.2. Animal characteristics

By far, the strongest association in our models with both the act of bringing an animal for testing and a rabies positive test was the animal health appearance assigned by the doctor based on symptoms and behaviors described by the patient. This variable correctly predicted the positive rabid status of 93.4% of animals that were tested. Removing this variable led to a model with a lower predictive performance. This suggests that the medical doctors who conducted the interview were very proficient at correctly identifying potential rabid animals based on the symptoms and behaviors reported by the patients. Unfortunately, not all indicators used to assign this health appearance were individually recorded in the patient surveys, notably observed symptoms, and it could be useful for future studies to record these individually to identify which are most predictive of a positive animal. Studies in Thailand and the Philippines looking at behaviors and symptoms more specifically found that the most predictive factors of a rabies positive animal included behavioral changes. Typical rabies symptoms such as salivation and paralysis were also associated with positive animals, but with a lower effect size [26,44].

More specifically, in our study, unprovoked aggression was more likely to come with a positive test than provoked aggression: aggressiveness is one of the oldest and most commonly described behavioral symptoms in rabid animals [45–47]. This is in agreement with results in Thailand and the Philippines [26,44]. Furthermore, the likelihood of a positive rabies test increased significantly with the number of victims: a rabid, thus more aggressive, animal is more likely to attack multiple individuals at random, whereas healthy animals will target aggression to the individual responsible for the provocation. These variables were similarly predictive of the biting animal being brought to IPC, suggesting good awareness and rabies knowledge in the PEP patient population of the risks associated with aggressive dogs.

Nearly 90% of tested animals were owned. Furthermore, 98% of PEP patients reported that the attacking animal was owned, regardless of whether the animal was tested, indicating that stray dogs are uncommon in Cambodia, which has also been observed in demographic studies [9]. Animals for which the owner was not identified were much more likely to be positive. This can probably be explained by the fact that rabid animals present erratic behavior once symptoms start, they stray away from home and attack in locations other than where they could have been identified by their owner. At the population level, it was observed in Thailand that districts with higher number of stray dogs were more likely to have rabies [48].

The vast majority of tested animals were dogs (98%). Despite their low number, cats were significantly less likely to test positive; this result was also observed in two studies from Thailand, where around 15% of cats were positive compared to more than 50% of dogs, with larger numbers of cats tested [26,42]. Although the results observed for livestock were not statistically significant, we observed a higher positive rate in livestock than dogs. Similarly, 73% (138/189) of cattle in one of the Thai studies tested positive [26]. Though this does not allow for definitive conclusions on livestock rates of rabies infection, it does provide evidence that rabies infection is not negligible in livestock species, which should be more considered in public awareness campaigns. The low proportion of PEP patients coming for livestock exposure (0.26%), and the lower odds of these being tested suggests that there is much lower awareness of the risks of rabies infection through livestock species compared to the awareness of dog aggression. Furthermore, species such as swine, cattle, or buffaloes are more complicated and expensive for individuals to transport for testing, especially if longer distances are involved.

### 4.3. Wound

Although wound severity and presence of wounds at the head and neck are used as one of the main indicators to guide PEP allocation and regimen according to WHO guidelines [14], our analysis did not show any evidence of association with the rabies test outcome of the animal. Nevertheless, they should remain an important factor in PEP allocation as this relates more to the transmission mechanism than the probability of the animal being rabid. Severe or deep wounds have increased risk of transmission should the animal be rabid as the initial steps of transmission require the transfer of saliva into muscle tissue where the virus will replicate before moving to the nervous system, whereas wounds to head have the shortest progression path from exposure site to the brain [1,4].

As has been observed in many other studies, the most common wound locations in IPC patients were feet, legs, and hands and fingers [27,28,49,50]. This is expected as lower limbs are the most immediately exposed parts of our body to a potential bite, whereas hands serve as our primary means of defense should an animal attack. Less intuitive is the evidence showing that wounds to the hands and arm were more likely to be associated with a rabid animal than wounds to the feet and legs. This could be due to the fact that healthy dogs will focus a quick aggression to easily accessible organs, whereas rabid dogs attack more persistently and indiscriminately.

For all of these variables, the association with the act of bringing an animal for testing indicated a similar interpretation. The odds of testing increased with severe wounds, the number of lesions, and wounds located to the head and neck. These suggest that perceptions of severity are associated with perceptions of risk and the decision-making process of bite victims.

### 4.4. Patient demographics

Approximately half (49%) of patients were children under the age of 14, which comprised 34% of the Cambodian population in the 2008 census [51]. This trend has also been observed in Thailand, Vietnam, and the Central African Republic [27,28,49]. However, children were also significantly less likely to have been attacked by a rabid animal compared to the adult age groups whilst being more likely to have an animal tested. One possible reason is that children might be more likely to provoke a healthy dog through playfulness and lack of awareness of the dog’s potential reaction, making them more likely to be bitten by healthy animals and then come to IPC for PEP. Furthermore, the risk perception threshold of a parent is often lower children then themselves, with adults possibly more likely to bring their child in lower risk situations.

### 4.5. Spatial distribution of rabies risk

As discussed in a prior paper, rates and numbers of people seeking PEP was strongly associated with the travel time to IPC in Phnom Penh, with five provinces being identified as most suitable for new center openings: Banteay Meanchey, Siem Reap, Svay Rieng, Takeo, and Kampot [23]. However, these provinces all had high odds of testing but lower odds of positive animals in this study. The two provinces of Kampong Cham and Kandal stood out in having the highest odds of exposure to a rabid animal, followed by Battambang and Kampong Thom, making these four provinces good candidates for PEP centers. As described in Section 4.1, two of these, Battambang and Kampong Cham have been the focus of new PEP center openings in 2018 and 2019. Furthermore, Battambang neighbors two provinces highlighted in our prior study on accessibility (Beanteay Meanchey and Siem Reap), highlighting a need in the North-West of Cambodia.

### 4.6. Limitations

One of the main limitations of this study came from the biased nature of the data resulting from passive voluntary reporting. Two forms of selection biases were observed. Firstly, PEP patients coming to IPC were biased by their accessibility to IPC as has been demonstrated in a previous paper [23]. Thus, patients and the animals they bring for testing are not a representative sample of the nation as a whole. Secondly, as demonstrated in Model 1, when patients do come to IPC, the perceived severity of the attack and the awareness about rabies influences the act of bringing an animal for testing. Thus, awareness of rabies and accessibility to a PEP center are important factors in the odds of a biting animal being tested for rabies. However, as the goal of patient screening and animal testing at IPC is to help identify the most at-risk patients and not establish disease burden in the canine population, this second selection bias should be seen as to an extent by design and desirable, as it shows a more resource-effective risk-based testing program, even if this is a limitation for modeling exercises. When comparing the models described in this paper, two patterns emerge. In many of the variables, Models 1 and 2 have similar results (i.e., the same direction of effect for the same category), leading to conclusion that in these cases patients correctly identify elements associated with rabies. In other cases, we see an inverse pattern. This can be seen most clearly with animal species, geography, and age (with livestock and certain provinces having lower odds of being tested but higher odds of being positive, or children showing the opposite trend). This likely reflects knowledge gaps and biased risk perception thresholds leading to overrepresentation of lower risk profiles and underrepresentation of higher risk profiles. Furthermore, variables that were predictive of bringing an animal for testing might also be predictive of a patient coming for PEP following an animal related injury in the first place, based on the perceived severity of the event and the general population awareness of rabies in general. We could hypothesize that individuals suffering from livestock related attacks are not only less likely to have the animal tested, but also less likely to seek treatment due to lower awareness or the more rural and distant setting in which livestock related events are likely to occur. Finally, the PEP patient population might represent a subset of the population with higher awareness of rabies in general.

Our models were also highly influenced by a single variable, and removing this variable led to a much less statistically robust model with very different and more uncertain predictions. Furthermore, this variable was an aggregate of a number primary variable which were unfortunately, not all available to us for modelling individually. Questions regarding individual symptoms of animals were not recorded in the dataset, whilst the question on the post-attack status of the animal was not usable as it is directly correlated to the fact the dog is available for testing. We conclude that though these models were good at identifying specific variables associated with rabies test outcomes, they were limited in terms of predicting exposure in patients without a tested animal. This resulted in fitted probabilities that either mostly aligned with the distribution of the animal health appearance variable or had very large credibility intervals when that variable was absent.

## 5. Conclusion

We provide strong evidence that rabies remains a major public health concern in Cambodia, despite the difficulty establishing a direct human burden of rabies in Cambodia. In an effort to establish predictors of rabies positive tests to guide PEP allocation, we observed that IPC already has a robust and highly sensitive and specific protocol to identify animals suspected of being rabid. Although we did identify specific predictors, these only marginally improved the performance of the protocol already used by IPC doctors. We also established a geographical distribution of the risk of rabies exposure. Notably Phnom Penh, which had the lowest uncertainty, had much lower risk of rabies exposure compared to other more rural provinces. Amongst provinces with large numbers of tested animals, we mostly saw a North-South divide, with coastal southern provinces less at risk and provinces north of Phnom Penh more at risk. However, the geographically biased nature of the data limits our interpretation of the spatial distribution of rabies. With new centers being opened by IPC in Kampong Cham and Battambang Provinces, there will likely be an improvement in the reach of animal testing in Cambodia leading to better identification of high-risk areas. However, beyond expanding PEP capacity and the passive surveillance it provides, which has been the main focus to date, there is a key need in Cambodia in terms of field investigation of rabies presence and developing a framework for canine vaccination in rural provinces to reduce the risk of exposure in humans.

## Supporting information

S1 File Questionnaire completed by IPC doctors when interviewing PEP patients on their first visit (document provided by IPC). (PDF)

S2 File Decision tree to assess the rabies status of the biting animal and to inform the allocation of RIG for PEP patients (document provided by IPC). (PDF)

S1 Table General summaries for counts and univariate analysis of all variables included in model selection. (DOCX)

S2 Table General summaries for variables used as random effects in multivariate model selections. (DOCX)

S3 Table Number of tested animals per province and exponentiated outputs from random effects of models 1 and 2. Odds ratios use the median parameter value from the parameter distribution.
(DOCX)

S4 Table Results from model 2 selection when removing the animal health appearance variable from the process. (DOCX)

S5 Table Patient numbers at IPC 2018–2020: This is the period covering the opening of new centers in Battambang and Kampong Cham until the beginning of the COVID Pandemic. (DOCX)
