# Supplementary material for: Analysis of predictors of rabies-positive biting animals in Cambodia using spatio-temporal Bayesian regression modelling
Source: PLoS Negl Trop Dis. 2025 Sep 5;19(9):e0013478. doi: 10.1371/journal.pntd.0013478 (PMC12431645; doi:10.1371/journal.pntd.0013478)
Supplement: S2 File — (PDF) [file pntd.0013478.s002.pdf]

Status of animal	Monitoring	Signs in animals<br>Sick/suspected
**Accessible**	Evaluating the circumstance of animal at time of bitten ➔ **Check signs**<br>Observation animal for 10 days	1- In a state of illness<br>- Drop tail<br>- Hyper salivation,<br>-Not recognize the owner<br>-Loss of orientation<br>-Abnormally aggressive<br>-Bite even objects, paralysis<br>2- Spontaneous aggression<br>3- Wounded multiple victims (human and animal)
**Dead of culling (slaughter)**	Evaluating the circumstance of animal at time of bitten ➔ **Check signs**<br>If animal head available for lab testing ➔ **Treatment bases on lab result** of rabies analysis<br>If animal head not available ➔ **Treatment bases on circumstance of animal**	
**Animal disappeared**	Evaluating the circumstance of animal at time of bitten ➔ **Check signs**	
**Dead of illness**	**Considering suspicious rabies animal**	

Revision on
23 Oct 2020

Diagram summarizing Prescription of Rabies Post-Exposure Prophylaxis (PEP) at the Rabies Prevention Center, Institut Pasteur du Cambodge (IPC)

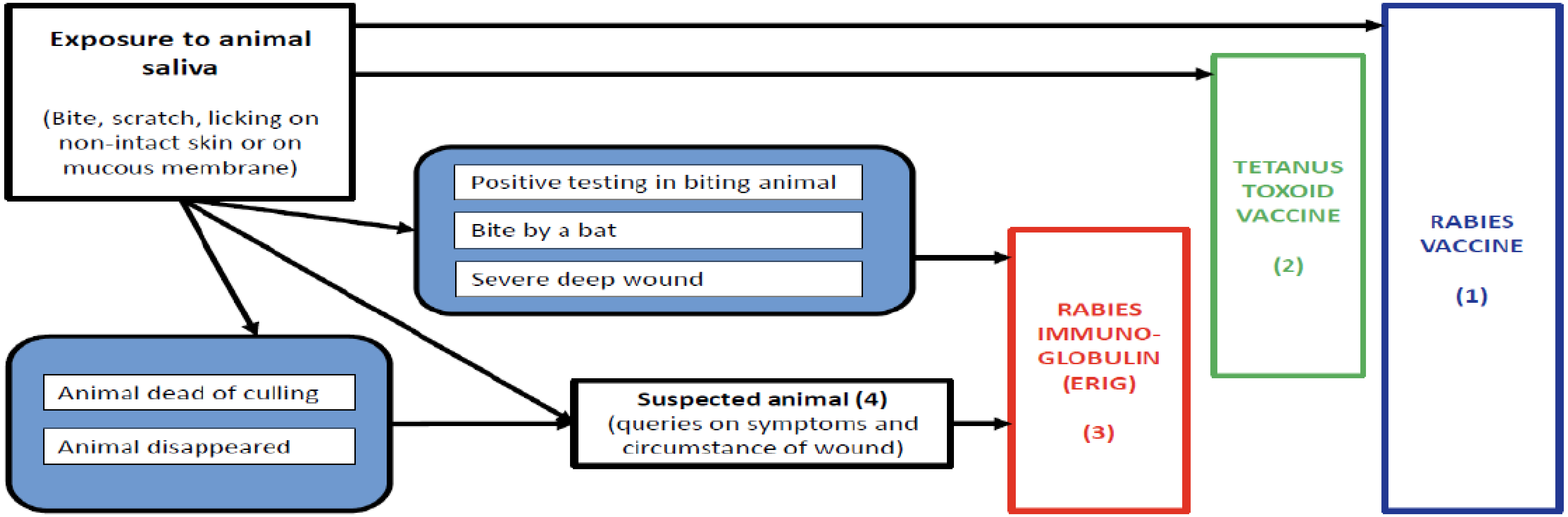

Supplementary Document SD2: Decision tree to assess the rabies status of the biting animal and to inform the allocation of RIG for PEP patients (document provided by IPC).
