# Supplementary material for: Analysis of predictors of rabies-positive biting animals in Cambodia using spatio-temporal Bayesian regression modelling
Source: PLoS Negl Trop Dis. 2025 Sep 5;19(9):e0013478. doi: 10.1371/journal.pntd.0013478 (PMC12431645; doi:10.1371/journal.pntd.0013478)
Supplement: S1 Table — (DOCX) [file pntd.0013478.s003.docx]

***S1 Table: General summaries for counts and univariate analysis of all variables included in model selection.***

| Variable | Category | Number of patients | Number of tests | Number of positives | OR for testing  (95% CI) | OR for test result  (95% CI) |
| --- | --- | --- | --- | --- | --- | --- |
| **Outcome variables** |  |  |  |  |  |  |
| Tested animals | No | 289,525 | - | - | - | - |
|  | Yes | 4,515 | - | - | - | - |
|  | Missing answer | 0 | - | - | - | - |
| Test results | No | - | 1,783 |  | - | - |
|  | Yes | - | 2,726 |  | - | - |
|  | Missing answer (inconclusive test results) | - | 6 | - | - | - |
| **Patient demographics** |  |  |  |  |  |  |
| Sex | Male | 153,060 | 2,511 | 1,570 | Ref | Ref |
|  | Female | 140,964 | 2,004 | 1,156 | 0.85 (0.80 to 0.90) | 0.83 (0.73 to 0.94) |
|  | Missing answer | 16 | 0 | 0 |  |  |
| Age | 0 to 9 years | 104,959 | 1,730 | 917 | Ref | Ref |
|  | 10 to 19 years | 62,351 | 981 | 624 | 1.01 (1.01 to 1.19) | 1.62 (1.37 to 1.90) |
|  | 20 to 59 years | 112,519 | 1,619 | 1,058 | 1.00 (0.94 to 1.07) | 1.69 (1.47 to 1.95) |
|  | 60 years or above | 14,186 | 185 | 127 | 0.88 (0.76 to 1.02) | 1.97 (1.41 to 2.74) |
|  | Missing answer | 25 | 0 | 0 |  |  |
| **Exposure and wound characteristics** |  |  |  |  |  |  |
| Time from accident to consultation | 1 day | 185,321 | 3,099 | 1,965 | Ref | Ref |
|  | 2 or 3 days | 77,721 | 983 | 510 | 0.53 (0.50 to 0.57) | 0.65 (0.56 to 0.75) |
|  | 4 or 5 days | 21,047 | 281 | 159 | 0.47 (0.42 to 0.54) | 0.80 (0.62 to 1.03) |
|  | 6 days or more | 9,852 | 152 | 92 | 0.48 (0.41 to 0.57) | 0.94 (0.67 to 1.32) |
|  | Missing answer | 99 | 0 | 0 |  |  |
| Exposure type | Bite | 292,414 | 4,499 | 2,717 | Ref | Ref |
|  | Other | 1,618 | 14 | 9 | 0.33 (0.21 to 0.51) | 1.13 (0.37 to 3.44) |
|  | Missing answer | 8 | 2 | 0 |  |  |
| Number of victims | 1 | 231,864 | 2,446 | 1,226 | Ref | Ref |
|  | 2 or 3 | 48,485 | 1,431 | 981 | 2.87 (2.68 to 3.06) | 2.13 (1.85 to 2.45) |
|  | 4 or 5 | 10,115 | 383 | 304 | 3.79 (3.40 to 4.22) | 3.77 (2.89 to 4.90) |
|  | 6 or more | 3,523 | 252 | 214 | 7.32 (6.41 to 8.36) | 5.80 (4.05 to 8.30) |
|  | Missing answer | 53 | 3 | 1 |  |  |
| Contact surface | Healthy skin | 293,619 | 4,503 | 2,720 | Ref | Ref |
|  | Damaged skin | 222 | 10 | 0 | 2.17 (1.51 to 3.12) | 0.85 (0.23 to 3.09) |
|  | Mucosa | 27 | 0 | 0 | - | - |
|  | Missing answer | 172 | 2 | 0 |  |  |
| Severity of wound | Superficial | 247,083 | 3,382 | 2,052 | Ref | Ref |
|  | Deep | 46,748 | 1,131 | 674 | 1.63 (1.53 to 1.75) | 0.91 (0.79 to 1.04) |
|  | Missing answer | 209 | 2 | 0 |  |  |
| Interposition of clothes | Yes | 98,991 | 1,236 | 705 | Ref | Ref |
|  | No | 194,833 | 3,277 | 2,021 | 1.26 (1.18 to 1.34) | 1.24 (1.09 to 1.43) |
|  | Missing answer | 216 | 2 | 0 |  |  |
| Number of lesions | 1 | 48,624 | 541 | 335 | Ref | Ref |
|  | 2 | 221,608 | 3,261 | 1,968 | 1.03 (0.95 to 1.13) | 0.94 (0.77 to 1.14) |
|  | 3 | 16,595 | 426 | 265 | 1.77 (1.56 to 2.01) | 0.95 (0.72 to 1.24) |
|  | 4 | 4,791 | 172 | 103 | 2.83 (2.39 to 3.36) | 0.88 (0.61 to 1.26) |
|  | 5 or more | 2,171 | 112 | 55 | 3.77 (3.08 to 4.62) | 0.52 (0.34 to 0.80) |
|  | Missing answer | 247 | 3 | 0 |  |  |
| Location of wound (dummy variables) |  |  |  |  |  |  |
| *Head and neck* | No | 275,891 | 4,110 | 2,520 | Ref | Ref |
|  | Yes | 17,929 | 402 | 205 | 1.41 (1.27 to 1.56) | 0.63 (0.51 to 0.78) |
|  | Missing answer | 220 | 3 | 1 |  |  |
| *Trunk and genitals* | No | 266,491 | 4,094 | 2,497 | Ref | Ref |
|  | Yes | 27,329 | 418 | 228 | 1.04 (0.94 to 1.15) | 0.73 (0.59 to 0.89) |
|  | Missing answer | 220 | 3 | 1 |  |  |
| *Arms* | No | 277,717 | 4,224 | 2,546 | Ref | Ref |
|  | Yes | 16,103 | 268 | 179 | 1.40 (1.24 to 1.58) | 1.30 (0.99 to 1.70) |
|  | Missing answer | 220 | 3 | 1 |  |  |
| *Hands and fingers* | No | 236,422 | 3,382 | 1,997 | Ref | Ref |
|  | Yes | 57,398 | 1,130 | 728 | 1.52 (1.42 to 1.63) | 1.29 (1.12 to 1.49) |
|  | Missing answer | 220 | 3 | 1 |  |  |
| *Legs* | No | 212,034 | 3,516 | 2,137 | Ref | Ref |
|  | Yes | 81,786 | 996 | 588 | 0.82 (0.77 to 0.88) | 0.91 (0.79 to 1.06) |
|  | Missing answer | 220 | 3 | 1 |  |  |
| *Feet* | No | 193,906 | 2,998 | 1,798 | Ref | Ref |
|  | Yes | 99,914 | 1,514 | 927 | 0.80 (0.75 to 0.85) | 1.08 (0.95 to 1.23) |
|  | Missing answer | 220 | 3 | 1 |  |  |
| **Animal characteristics** |  |  |  |  |  |  |
| Species | Dog | 277,629 | 4,428 | 2,686 | Ref | Ref |
|  | Cat | 12,745 | 45 | 12 | 0.25 (0.19 to 0.33) | 0.21 (0.10 to 0.41) |
|  | Livestock | 769 | 35 | 27 | 1.67 (1.25 to 2.22) | 2.10 (0.94 to 4.68) |
|  | Wild animals | 2,651 | 7 | 1 | 0.19 (0.11 to 0.32) | 0.10 (0.01 to 0.81) |
|  | Human exposure or prevention | 20 | 0 | 0 | - | - |
|  | Missing answer | 226 | 0 | 0 |  |  |
| Aggression type | Spontaneous | 195,672 | 3,406 | 2,190 | Ref | Ref |
|  | Provoqued | 98,142 | 1,107 | 536 | 0.71 (0.67 to 0.76) | 0.53 (0.46 to 0.61) |
|  | Missing answer | 226 | 2 | 0 |  |  |
| Health appearance | Healthy | 285,991 | 1,789 | 150 | Ref | Ref |
|  | Sick | 7,833 | 2,726 | 2,576 | 55.64 (51.95 to 59.60) | 257.28 (196.47 to 336.90) |
|  | Missing answer | 216 | 0 | 0 |  |  |
| Ownership | With owner | 288,527 | 4,043 | 2,276 | Ref | Ref |
|  | Without owner | 5,291 | 471 | 450 | 5.39 (4.87 to 5.96) | 16.87 (10.82 to 26.29) |
|  | Missing answer | 222 | 1 | 0 |  |  |
