# Supplementary material for: Analysis of predictors of rabies-positive biting animals in Cambodia using spatio-temporal Bayesian regression modelling
Source: PLoS Negl Trop Dis. 2025 Sep 5;19(9):e0013478. doi: 10.1371/journal.pntd.0013478 (PMC12431645; doi:10.1371/journal.pntd.0013478)
Supplement: S2 Table — (DOCX) [file pntd.0013478.s004.docx]

***S2 Table: General summaries for variables used as random effects in multivariate model selections.***

| Variable | Category | Number of patients | Number of tests | Number of positives |
| --- | --- | --- | --- | --- |
| Year of exposure | 2000 | 11,899 | 143 | 97 |
|  | 2001 | 12,518 | 156 | 103 |
|  | 2002 | 12,941 | 214 | 160 |
|  | 2003 | 12,121 | 154 | 84 |
|  | 2004 | 13,085 | 212 | 130 |
|  | 2005 | 13,637 | 153 | 88 |
|  | 2006 | 13,363 | 175 | 89 |
|  | 2007 | 14,593 | 268 | 157 |
|  | 2008 | 20,334 | 412 | 257 |
|  | 2009 | 22,050 | 471 | 291 |
|  | 2010 | 21,030 | 357 | 180 |
|  | 2011 | 20,038 | 332 | 183 |
|  | 2012 | 20,609 | 362 | 225 |
|  | 2013 | 21,075 | 345 | 215 |
|  | 2014 | 21,782 | 321 | 198 |
|  | 2015 | 21,301 | 238 | 147 |
|  | 2016 | 21,664 | 202 | 122 |
|  | Missing answer | 0 | 0 | 0 |
| Month of exposure | 1 | 24,864 | 405 | 270 |
|  | 2 | 24,219 | 416 | 278 |
|  | 3 | 26,429 | 434 | 268 |
|  | 4 | 23,621 | 323 | 189 |
|  | 5 | 22,956 | 357 | 208 |
|  | 6 | 23,168 | 348 | 204 |
|  | 7 | 24,084 | 357 | 197 |
|  | 8 | 25,355 | 413 | 250 |
|  | 9 | 23,978 | 345 | 184 |
|  | 10 | 25,107 | 349 | 209 |
|  | 11 | 24,339 | 346 | 199 |
|  | 12 | 25,920 | 422 | 270 |
|  | Missing answer | 0 | 0 | 0 |
| Accident location (province) | KH01 Banteay Mean Chey | 229 | 23 | 15 |
|  | KH02 Battambang | 507 | 9 | 8 |
|  | KH03 Kampong Cham | 22,809 | 655 | 474 |
|  | KH04 Kampong Chhnang | 4,143 | 71 | 39 |
|  | KH05 Kampong Speu | 9,870 | 612 | 334 |
|  | KH06 Kampong Thom | 3,432 | 106 | 83 |
|  | KH07 Kampot | 4,465 | 443 | 236 |
|  | KH08 Kandal | 60,267 | 728 | 526 |
|  | KH09 Koh Kong | 291 | 10 | 8 |
|  | KH10 Kratie | 545 | 16 | 12 |
|  | KH11 Mondul Kiri | 46 | 0 | 0 |
|  | KH12 Phnom Penh | 158,009 | 544 | 285 |
|  | KH13 Preah Vihear | 67 | 2 | 2 |
|  | KH14 Prey Veaeng | 12,507 | 550 | 306 |
|  | KH15 Pursat | 435 | 3 | 3 |
|  | KH16 Ratanak Kiri | 66 | 3 | 1 |
|  | KH17 Siem Reap | 356 | 13 | 7 |
|  | KH18 Preah Sihanouk | 482 | 11 | 8 |
|  | KH19 Stueng Treng | 40 | 0 | 0 |
|  | KH20 Svay Rieng | 1,738 | 44 | 18 |
|  | KH21 Takaeo | 13,563 | 666 | 360 |
|  | KH22 Otdar Meanchey | 42 | 4 | 1 |
|  | KH23 Kep | 12 | 0 | 0 |
|  | KH24 Krong Pailin | 34 | 0 | 0 |
|  | Missing answer | 85 | 2 | 0 |
