# Supplementary material for: Analysis of predictors of rabies-positive biting animals in Cambodia using spatio-temporal Bayesian regression modelling
Source: PLoS Negl Trop Dis. 2025 Sep 5;19(9):e0013478. doi: 10.1371/journal.pntd.0013478 (PMC12431645; doi:10.1371/journal.pntd.0013478)
Supplement: S3 Table — Odds ratios use the median parameter value from the parameter distribution. (DOCX) [file pntd.0013478.s005.docx]

***S3 Table: Number of tested animals per province and exponentiated outputs from random effects of models 1 and 2. Odds ratios use the median parameter value from the parameter distribution.***

| Province | PEP patients | Tested Animals  (% of patients) | Positive Animals  (% positive) | Odds Ratio  Model 1  (95% CI) | Odds Ratio  Model 2  (95% CI) |
| --- | --- | --- | --- | --- | --- |
| TOTAL | 293,625 | 4,508 (1.5) | 2,724 (60.4) |  |  |
| KH01 Banteay Mean Chey | 229 | 23 (10.0) | 15 (65.2) | 1.84 (0.99 to 3.53) | 0.75 (0.29 to 1.75) |
| KH02 Battambang | 506 | 8 (1.6) | 8 (100.0) | 0.61 (0.30 to 1.23) | 1.27 (0.53 to 3.57) |
| KH03 Kampong Cham | 22,785 | 655 (2.9) | 474 (72.6) | 1.31 (0.93 to 1.89) | 1.91 (1.22 to 3.14) |
| KH04 Kampong Chhnang | 4,135 | 71 (1.7) | 39 (54.9) | 0.75 (0.50 to 1.16) | 1.02 (0.52 to 1.99) |
| KH05 Kampong Speu | 9,865 | 612 (6.2) | 334 (54.7) | 3.18 (2.26 to 4.62) | 0.91 (0.58 to 1.44) |
| KH06 Kampong Thom | 3,431 | 106 (3.1) | 83 (78.3) | 1.21 (0.82 to 1.83) | 1.28 (0.67 to 2.53) |
| KH07 Kampot | 4,458 | 443 (9.9) | 236 (53.3) | 4.38 (3.09 to 6.42) | 0.99 (0.61 to 1.62) |
| KH08 Kandal | 60,235 | 728 (1.2) | 526 (72.3) | 0.86 (0.61 to 1.24) | 1.63 (1.03 to 2.67) |
| KH09 Koh Kong | 290 | 10 (3.4) | 8 (80.0) | 0.88 (0.42 to 1.85) | 1.06 (0.40 to 2.94) |
| KH10 Kratie | 545 | 16 (2.9) | 12 (75.0) | 0.77 (0.42 to 1.40) | 1.10 (0.45 to 2.61) |
| KH11 Mondul Kiri | 46 | 0 | 0 | 0.57 (0.14 to 2.11) | 1.06 (0.35 to 3.12) |
| KH12 Phnom Penh | 157,820 | 544 (0.3) | 285 (52.4) | 0.35 (0.25 to 0.50) | 0.30 (0.19 to 0.48) |
| KH13 Preah Vihear | 67 | 2 (3.0) | 2 (100.0) | 0.77 (0.25 to 2.27) | 1.04 (0.39 to 2.79) |
| KH14 Prey Veaeng | 12,481 | 548 (4.4) | 305 (55.9) | 2.38 (1.69 to 3.46) | 0.93 (0.58 to 1.49) |
| KH15 Pursat | 435 | 3 (0.7) | 3 (100.0) | 0.22 (0.09 to 0.50) | 1.06 (0.41 to 2.85) |
| KH16 Ratanak Kiri | 66 | 3 (4.5) | 1 (33.3) | 0.89 (0.30 to 2.50) | 0.94 (0.29 to 2.79) |
| KH17 Siem Reap | 351 | 13 (3.7) | 7 (53.8) | 0.66 (0.35 to 1.23) | 0.91 (0.36 to 2.12) |
| KH18 Preah Sihanouk | 470 | 11 (2.3) | 8 (72.7) | 0.69 (0.35 to 1.31) | 1.08 (0.42 to 2.94) |
| KH19 Stueng Treng | 40 | 0 | 0 | 0.66 (0.16 to 2.45) | 1.06 (0.39 to 2.84) |
| KH20 Svay Rieng | 1,733 | 44 (2.5) | 18 (40.9) | 1.71 (1.07 to 2.76) | 0.78 (0.30 to 1.91) |
| KH21 Takaeo | 13,549 | 664 (4.9) | 359 (54.1) | 2.90 (2.06 to 4.21) | 0.93 (0.59 to 1.48) |
| KH22 Otdar Meanchey | 42 | 4 (9.5) | 1 (25.0) | 2.26 (0.74 to 7.65) | 0.95 (0.33 to 2.58) |
| KH23 Kep | 12 | 0 | 0 | 0.74 (0.15 to 3.21) | 1.00 (0.27 to 3.74) |
| KH24 Krong Pailin | 34 | 0 | 0 | 0.56 (0.09 to 2.70) | 1.06 (0.28 to 4.29) |
