# Supplementary material for: Analysis of predictors of rabies-positive biting animals in Cambodia using spatio-temporal Bayesian regression modelling
Source: PLoS Negl Trop Dis. 2025 Sep 5;19(9):e0013478. doi: 10.1371/journal.pntd.0013478 (PMC12431645; doi:10.1371/journal.pntd.0013478)
Supplement: S4 Table — (DOCX) [file pntd.0013478.s006.docx]

***S4 Table: Results from model 2 selection when removing the animal health appearance variable from the process.***

| Variable | Category | Number of  tested animals | Number of  positive animals | Percentage  Of positive animals | Odds ratio  (Model 2B) | 95% credibility  interval |
| --- | --- | --- | --- | --- | --- | --- |
| Total | NA | 4,502 | 2,724 | 60.5 | NA | NA |
| **Animal** |  |  |  |  |  |  |
| Animal health appearance | Healthy | 1,783 | 150 | 8.4 | Removed from selection | |
|  | Sick | 2,719 | 2,574 | 94.7 |  |  |
| Aggression | Spontaneous | 3,400 | 2,188 | 64.35 | ref | - |
|  | Provoked | 1,102 | 536 | 48.6 | 0.50 | 0.42 to 0.59 |
| Animal ownership | Owned | 4,031 | 2,274 | 56.4 | ref | - |
|  | Feral or wild | 471 | 450 | 95.5 | 20.98 | 13.15 to 33.50 |
| Animal species | Dog | 4,416 | 2,684 | 60.8 | ref | - |
|  | Cat | 45 | 12 | 26.7 | 0.17 | 0.07 to 0.37 |
|  | Livestock | 34 | 27 | 79.4 | 2.26 | 0.89 to 5.75 |
|  | Wild | 7 | 1 | 14.3 | 0.04 | 0.00 to 0.43 |
| **Victim** |  |  |  |  |  |  |
| Sex | Male | 2502 | 1569 | 62.7 | ref | - |
|  | Female | 2000 | 1155 | 57.8 | 0.82 | 0.71 to 0.94 |
| Age categories | 0 to 9 years | 1,724 | 916 | 53.1 | Ref |  |
|  | 10 to 19 years | 978 | 624 | 63.8 | 1.53 | 1.27 to 1.84 |
|  | 20 to 59 years | 1,615 | 1,057 | 65.5 | 1.74 | 1.47 to 2.05 |
|  | 60 years or more | 185 | 127 | 68.7 | 1.84 | 1.26 to 2.69 |
| **Attack and wounds** |  |  |  |  |  |  |
| Days from accident  to consultation | 1 day | 3093 | 1963 | 63.5 | ref | - |
|  | 2 or 3 days | 977 | 510 | 52.2 | 0.60 | 0.50 to 0.71 |
|  | 4 or 5 days | 280 | 159 | 56.8 | 0.64 | 0.47 to 0.85 |
|  | 6 or more days | 152 | 92 | 60.5 | 0.73 | 0.49 to 1.08 |
| Number of victims | 1 | 2,438 | 1,225 | 50.3 | ref | - |
|  | 2 or 3 | 1,430 | 981 | 68.6 | 2.27 | 1.95 to 2.66 |
|  | 4 or 5 | 383 | 304 | 79.4 | 4.65 | 3.48 to 6.21 |
|  | 6 or more | 251 | 214 | 85.2 | 6.16 | 4.09 to 9.27 |
| Number of lesions | 1 | 538 | 335 | 62.3 | ref | - |
|  | 2 | 3,255 | 1,967 | 60.4 | 0.86 | 0.68 to 1.08 |
|  | 3 | 426 | 265 | 62.2 | 0.78 | 0.57 to 1.07 |
|  | 4 | 172 | 103 | 59.9 | 0.63 | 0.41 to 0.95 |
|  | 5 or more | 111 | 54 | 48.7 | 0.31 | 0.19 to 0.51 |
| Wound on hands  or fingers | no | 3375 | 1996 | 59.1 | ref | - |
|  | yes | 1127 | 728 | 64.6 | 1.77 | 1.45 to 2.13 |
| Wound on arms | no | 4235 | 2545 | 60.1 | ref | - |
|  | yes | 267 | 179 | 67.0 | 1.67 | 1.22 to 2.28 |
| Wound on legs | no | 3508 | 2136 | 60.9 | ref | - |
|  | yes | 994 | 588 | 59.2 | 0.82 | 0.68 to 0.99 |
| Wound on  trunk and genitals | no | 4084 | 2496 | 61.1 | ref | - |
|  | yes | 418 | 228 | 54.6 | 0.90 | 0.63 to 1.03 |
