# Supplementary material for: Analysis of predictors of rabies-positive biting animals in Cambodia using spatio-temporal Bayesian regression modelling
Source: PLoS Negl Trop Dis. 2025 Sep 5;19(9):e0013478. doi: 10.1371/journal.pntd.0013478 (PMC12431645; doi:10.1371/journal.pntd.0013478)
Supplement: S5 Table — (DOCX) [file pntd.0013478.s007.docx]

***S5 Table: Patient numbers at IPC 2018-2020: This is the period covering the opening of new centers in Battambang and Kampong Cham until the beginning of the COVID Pandemic.***

| Year | Month | Phnom Penh | Battambang | Kampong Cham |
| --- | --- | --- | --- | --- |
| 2018 | 01 | 1,955 | - | - |
|  | 02 | 1,920 | - | - |
|  | 03 | 2,097 | - | - |
|  | 04 | 1,877 | - | - |
|  | 05 | 1,970 | - | - |
|  | 06 | 1,782 | - | - |
|  | 07 | 1,910 | 5 | - |
|  | 08 | 2,120 | 79 | - |
|  | 09 | 2,032 | 131 | - |
|  | 10 | 2,193 | 198 | - |
|  | 11 | 2,319 | 188 | - |
|  | 12 | 2,906 | 600 | - |
| 2019 | 01 | 3,207 | 767 | - |
|  | 02 | 4,999 | 1,171 | - |
|  | 03 | 7,593 | 1,451 | 997 |
|  | 04 | 5,324 | 1,501 | 1,738 |
|  | 05 | 3,935 | 1,189 | 1,091 |
|  | 06 | 4,738 | 1,478 | 1,094 |
|  | 07 | 4,485 | 1,478 | 1,079 |
|  | 08 | 3,755 | 1,288 | 1,006 |
|  | 09 | 3,260 | 915 | 914 |
|  | 10 | 4,222 | 1,356 | 1,132 |
|  | 11 | 3,460 | 1,167 | 955 |
|  | 12 | 3,520 | 1,309 | 1,117 |
| 2020 | 01 | 3,811 | 1,240 | 1,108 |
|  | 02 | 3,008 | 1,007 | 950 |
|  | 03 | 2,488 | 922 | 706 |
|  | 04 | 1,959 | 770 | 575 |
|  | 05 | 2,437 | 820 | 586 |
|  | 06 | 2,843 | 975 | 703 |
|  | 07 | 3,206 | 1,145 | 737 |
|  | 08 | 2,643 | 976 | 675 |
|  | 09 | 2,760 | 986 | 719 |
|  | 10 | 3,081 | 1,208 | 817 |
|  | 11 | 3,119 | 1,293 | 912 |
|  | 12 | 3,279 | 1,615 | 1,105 |
